# *Bacillus velezensis* ZN-S10 Reforms the Rhizosphere Microbial Community and Enhances Tomato Resistance to TPN

**DOI:** 10.3390/plants12203636

**Published:** 2023-10-21

**Authors:** Enlei Chen, Shufen Chao, Bin Shi, Lu Liu, Mengli Chen, Yongli Zheng, Xiaoxiao Feng, Huiming Wu

**Affiliations:** 1 College of Advanced Agriculture Sciences, Zhejiang Agriculture and Forestry University, Hangzhou 310058, China; 2 Zhejiang Agricultural Products Green Development Center, Hangzhou 310003, China; 3 Agricultural Experiment Station, Zhejiang University, Hangzhou 310058, China

**Keywords:** tomato pith necrosis, *Bacillus velezensis*, *Pseudomonas viridiflava*, rhizosphere microbial

## Abstract

Tomato pith necrosis (TPN) is a highly destructive disease caused by species of the *Pseudomonas* genus and other bacteria, resulting in a significant reduction in tomato yield. Members of the genus *Bacillus* are beneficial microorganisms extensively studied in the rhizosphere. However, in most cases, the potential of *Bacillus* members in controlling TPN and their impact on the rhizosphere microbial composition remain rarely studied. In this study, *Bacillus velezensis* ZN-S10 significantly inhibited the growth of *Pseudomonas viridiflava* ZJUP0398-2, and ZN-S10 controlled TPN with control efficacies of 60.31%. *P. viridiflava* ZJUP0398-2 significantly altered the richness and diversity of the tomato rhizobacterial community, but pre-inoculation with ZN-S10 mitigated these changes. The correlation analysis revealed that ZN-S10 maybe inhibits the growth of nitrogen-fixing bacteria and recruits beneficial bacterial communities associated with disease resistance, thereby suppressing the occurrence of diseases. In summary, the comparative analysis of the rhizosphere microbiome was conducted to explore the impact of ZN-S10 on the composition of rhizosphere microorganisms in the presence of pathogenic bacteria, aiming to provide insights for further research and the development of scientific and eco-friendly control strategies for this disease.

## 1. Introduction

Tomato (*Solanum lycopersicum*) is an important vegetable cultivated in both greenhouse and field environments, and it has gained popularity due to its unique flavor and rich nutritional value [1,2]. According to the Food and Agriculture Organization Statistical Database, global tomato production reached 189 million tons, harvested from 5 million hectares of land, in 2021 [3]. Asia emerged as the primary region for tomato production, with an output of 119 million tons. China, in particular, claimed the top spot in terms of both tomato production and cultivated area worldwide [3]. In 2021, China’s tomato production exceeded 67 million tons (excluding data from Hong Kong, Macau, and Taiwan), establishing itself as the largest tomato-producing country globally. Europe followed as the second-largest production area, with an output of 24 million tons. Spain, Italy, and Turkey featured among the top ten tomato-producing nations worldwide [3]. Various tomato diseases pose a significant threat to the tomato industry, greatly hindering its development. Tomato pith necrosis (TPN) is a globally significant disease caused by the members of the *Pseudomonas* genus and other bacteria, resulting in severe reductions in tomato yield. Currently, seven species of the *Pseudomonas* genus have been reported to cause TPN in tomato: *Pseudomonas corrugate* [4], *Pseudomonas cichori* [5], *Pseudomonas mediterranea* [6,7], *Pseudomonas fluorescens* [8], *Pseudomonas putida* [9], *Pseudomonas marginalis* [9], and *Pseudomonas viridiflava* [10]. At present, chemical fungicides are primarily used for preventing and controlling TPN [1]. However, the overuse of chemical fungicides has been shown to have a negative impact on beneficial microbial communities and the environment [11]. For example, tebuconazole and difenoconazole can reduce the biomass and activity of soil microorganisms, thereby disrupting the structures of soil microbial communities [12,13].

The rhizosphere harbors abundant microbial communities and is considered the “second genome” of plants, playing a crucial role in the health of plants [14,15]. Introducing exogenous bacteria into the soil can significantly affect original ecosystems by modifying the community structure of soil rhizosphere microorganisms. These bacteria colonize plant roots, reaching a specific population density, or they can reside in plant roots for a specific period of time [16,17]. For example, combinations of beneficial microorganisms such as *Rhizobia*, *pseudomonads*, *Agrobacteria*, and lytic bacteriophages were recruited by *Bacillus* and *Trichoderma* spp. [18,19]. Simultaneously, the invasion of pathogens can also alter the diversity and composition of the rhizosphere microbiome [20]. *Ralstonia solanacearum* disrupts the rhizosphere bacterial microbiome during an invasion [20]. 

Members of the genus *Bacillus* are extensively studied beneficial microorganism in rhizospheres [21]. Due to its effectiveness in biological control, several commercial strains of *Bacillus* have been employed in agricultural production [22], such as *Bacillus velezensis* GB03, *Bacillus velezensis* QST 713, *Bacillus velezensis* FZB42, and *Bacillus velezensis* D747 [23]. The *Bacillus* species can directly reduce the damage caused by plant pathogens through competitive and antagonistic interactions [24,25,26]. Additionally, they can indirectly defend against plant pathogens and promote plant growth by assembling a self-serving rhizosphere microbe and inducing plants to produce resistant substances [22]. The biocontrol abilities of the *Bacillus* species and their promotion of plant growth have been extensively researched. However, the potential of controlling TPN and its influence on rhizosphere microbial composition under conditions of bacterial invasion, in most cases, remain relatively unexplored.

**Figure 1:**
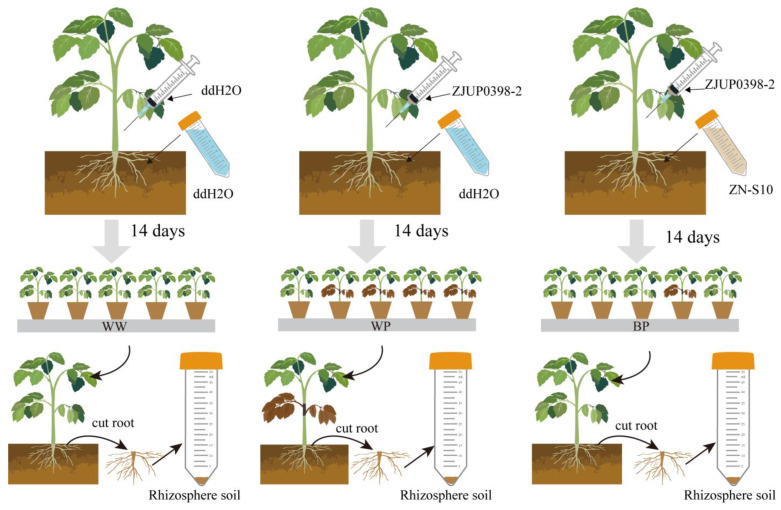
Schematic diagram. The tomato plants’ roots in the WP group were irrigated with sterile water. In the BP group, the roots were watered with *B. velezensis* ZN-S10 bacterial solution. After 7 days, both the WP and BP groups had *P. viridiflava* ZJUP0398-2 inoculated in the stem of the tomato plants. The WW group without the inoculation of ZN-S10 and *P. viridiflava* ZJUP0398-2 was set as control. After 14 days, it was advised to gather the rhizosphere soil for microbiome analysis.

Based on the aforementioned background, *Bacillus velezensis* exhibits significant potential for bio-control. It has been suggested that microbial inoculants can effectively suppress diseases and promote growth by enlisting beneficial species that modify the structure and function of the soil microbiome, in addition to their direct impact on the host plant [22]. Consequently, we hypothesize that *Bacillus velezensis* ZN-S10 can safeguard tomato plants against TPN by influencing the root microbiome. In this study, we examined the efficacy of the endophytic bacterium *B. velezensis* ZN-S10, which was isolated from tomato roots, in controlling tomato pith necrosis caused by *P. viridiflava*. Root irrigation with ZN-S10 was conducted to investigate its inhibitory effect in controlling TPN in tomatoes (Figure 1). Furthermore, a comparative analysis of the rhizosphere microbiome was performed to investigate the influence of ZN-S10 on the composition of rhizosphere microorganisms in the presence of pathogenic bacteria. The objective was to gain valuable insights for future research and for the formulation of environmentally friendly control strategies for this disease.

## 2. Results

### 2.1. Biocontrol Effect of ZN-S10 on TPN of Tomato

**Figure 2:**
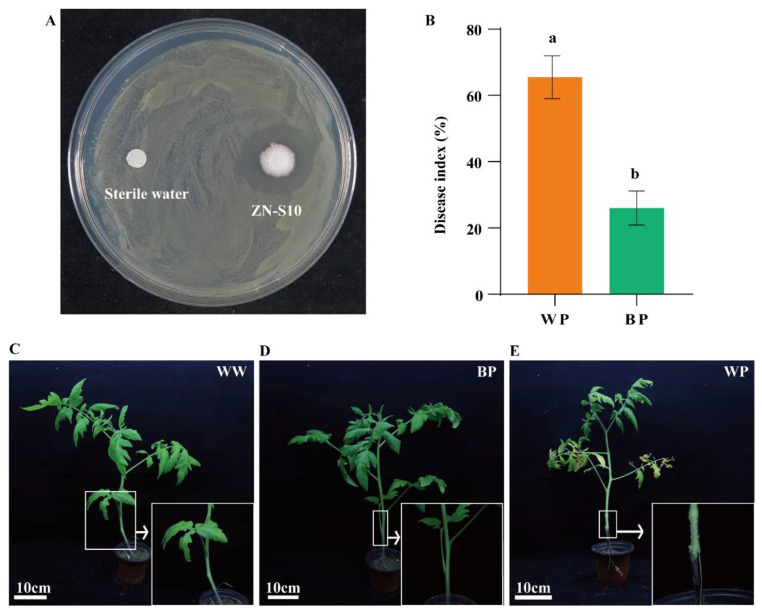
Control efficacy of *B. velezensis* ZN-S10 on TPN. (**A**) ZN-S10 inhibited the growth of ZJUP0398-2. “ZJUP-0398-2 was coated on a flat-surface, filter paper sheet with sterile water and ZN-S10”; (**B**) disease index for WW, BP, and WP. Growth statuses of tomatoes in different treatments. Different lowercase letters indicate significant differences between indices (*p* < 0.05, *t*-test). (**C**) The WW group without the inoculation of ZN-S10 and *P. viridiflava* ZJUP0398-2 was set as control. (**D**) The roots were watered with sterile water, and the stems were inoculated with *P. viridiflava* ZJUP0398-2. (**E**) The roots were watered with ZN-S10 bacterial solution, and the stems were inoculated with *P. viridiflava* ZJUP0398-2.

We isolated an antagonistic strain from tomato roots, named ZN-S10 (CP102933.1), and we also isolated the pathogenic bacterium from the infected tomato plants, named ZJUP0398-2. A phylogenetic tree based on the 16S rRNA gene was constructed using MEGA 6.0 [27] with neighbor-joining algorithms, which revealed that ZN-S10 is a member of the *Bacillus velezensis*, and ZJUP0398-2 is a species of *P. viridiflava*. The effect of ZN-S10 on the growth of the pathogenic bacterium ZJUP0398-2 was assessed by dual-culture assays. Notably, the isolate ZN-S10 inhibited the growth of ZJUP0398-2 (Figure 2A). The results suggested that ZN-S10 displayed strong inhibitory activity against ZJUP0398-2. In order to verify the antibacterial activity of ZN-S10, we conducted root irrigation with ZN-S10 on tomatoes. Tomato plants were grown in sterile soil inoculated (or not) with ZN-S10 (Figure 1). Disease incidence in the irrigation with ZN-S10 soil (BP) was 26.00% in the presence of ZJUP0398-2, whereas disease incidence in the irrigation without ZN-S10 soil (WP) exceeded 65.5% (Figure 2B, Table S1). The results suggested that ZN-S10 had a good biocontrol effect on tomato pith necrosis caused by ZJUP0398-2 (Figure 2C–E).

### 2.2. Sequencing and Taxonomic Assignments

High-throughput sequencing of marker genes (16S for bacteria and ITS for fungi) was conducted to assess the influence of ZN-S10 on the microbiome structure in the tomato rhizosphere. Rarefaction curves and rank abundance curves were utilized to assess the coverage depth of each sample. We observed that the rarefaction curves and rank abundance curves of 15 samples for 16S rDNA and ITS gene sequencing appeared to reach saturation, suggesting that the sequencing depths for these samples were adequate (Figures S1 and S2). A total of 193,182 high-quality sequences for 16S rDNA were retained in a range of 11,548 to 14,185 sequences per sample, and 189,152 high-quality sequences for ITS were retained in a range of 9373 to 14,779 sequences per sample (Table S2).

Taxonomic annotation of feature sequences was processed by Bayesian classifier and blast using SILVA as the bacterial reference database and UNITE as the fungal reference database, resulting in a total of 412 bacterial OTUs and 178 fungal OTUs, with a 97% similarity level. These OTUs belonged to 24 bacterial phyla and 11 fungal phyla. Among the OTUs, 509 OTUs, 445 OTUs, and 434 OTUs belonged to WW, WP, BP, respectively. A total of 399 OTUs were discovered among these three treatments. The obtained OTUs were devoted to deepening analyses to investigate the impact of ZN-S10 on the tomato rhizosphere microbiome.

### 2.3. Microbial Alpha Diversity Analysis

**Figure 3:**
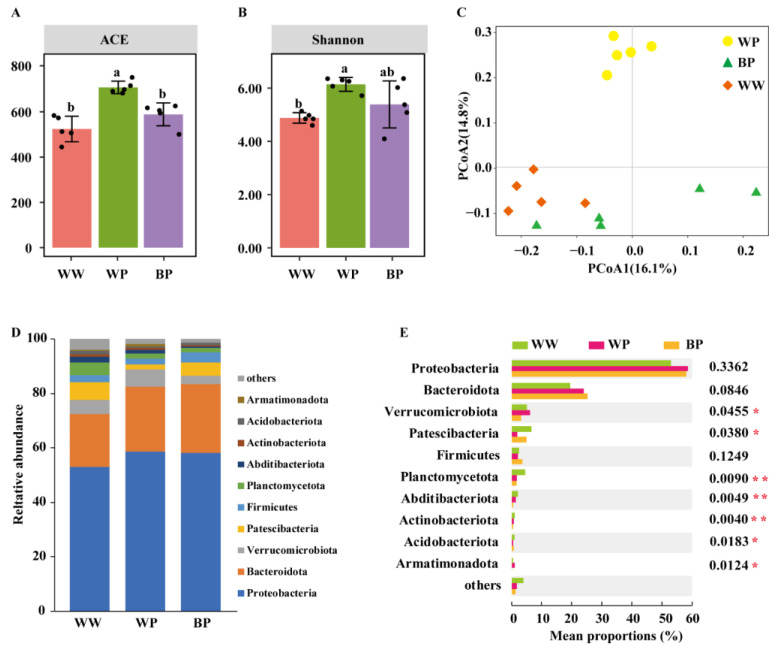
Bacterial alpha diversity, beta diversity, and microbiome composition analysis. Bacterial alpha diversity, as measured by ACE (**A**) or Shannon (**B**), varied among the rhizosphere soil samples from different treatments. Different lowercase letters indicate significant differences between indices (*p* < 0.05, Tukey test). Principal coordinate analysis (PCoA) using the abundance-based Bray–Curtis distance matrix (**C**) revealed distinct clustering patterns. The relative abundance of different phyla in the rhizosphere soil samples from plants of different treatments is shown in (**D**). The Kruskal–Wallis H test analysis (**E**) demonstrated significant differences in bacteria at the phylum level (* *p* < 0.05, ** *p* < 0.01).

**Figure 4:**
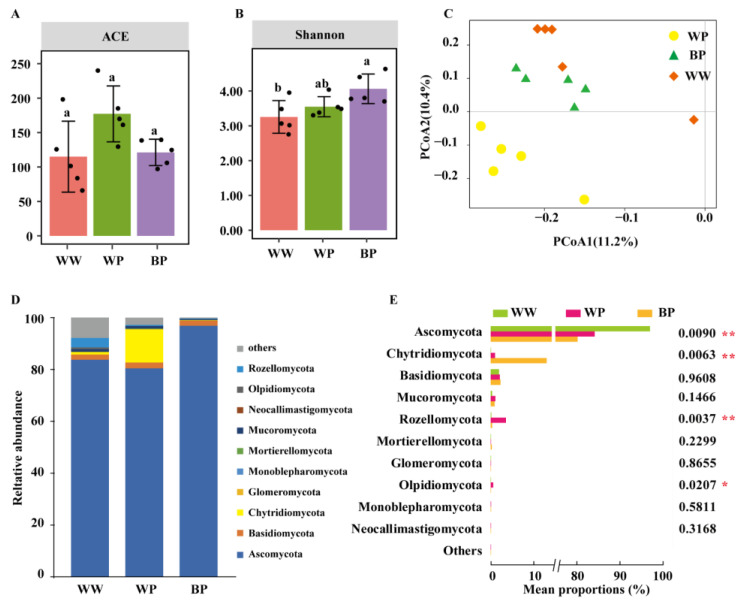
Fungal diversity and microbiome composition analysis of fungi. Fungal alpha diversity, as measured by ACE (**A**) or Shannon (**B**), varied among rhizosphere soil samples from different treatments. Different lowercase letters indicate significant differences between indices (*p* < 0.05, Tukey test). Principal coordinate analysis (PCoA) using the abundance-based Bray–Curtis distance matrix (**C**) revealed distinct clustering patterns. The relative abundances of different phyla in rhizosphere soil samples from plants of different treatments are shown in (**D**). The Kruskal–Wallis H test analysis (**E**) demonstrated significant differences in bacteria at the phylum level (* *p* < 0.05, ** *p* < 0.01).

Overall, there were significant differences in the bacterial community of the rhizosphere soil between different groups (*p* < 0.05, Figure 3A,B) but no significant differences in fungal community (*p* > 0.05, Figure 4A,B). The ACE index reflects species richness [28], while the Shannon index reflects species abundance and evenness [29]. The richness and diversity of the rhizosphere bacterial community of diseased plants that were inoculated with ZJUP0398-2 (WP) were significantly higher than the plants treated with water (WW) (*p* < 0.05, Figure 3A). Compare with WP, a significantly lower richness of the bacterial community was found in the samples that were treated with ZN-S10 and ZJUP0398-2 (BP) (*p* < 0.05, Figure 3B), and the diversity showed the same trend, although the difference was not significant (*p* > 0.05, Figure 3B). For the fungal community, the richness and diversity between WW and WP demonstrated a similar trend with the bacterial community. The richness of the fungal community between WP and BP showed a declining trend, but the diversity demonstrated an opposite trend; all these differences were not significant (*p* > 0.05, Figure 4A,B).

### 2.4. Beta Diversity and Community Composition of Bacteria

Beta diversity-based principal coordinate analysis (PCoA) of Bray–Curtis distances further revealed a significant difference in the bacterial community (Figure 3C). Samples that were treated with ZJUP0398-2 showed a tendency to cluster together, while samples inoculated with ZN-S10 and ZJUP0398-2 could be separated from samples only inoculated with ZJUP0398-2, but it seemed that samples in BP and WW could not be separated. We also identified these cluster results through the beta diversity analysis of Jaccard distance, resulting in a more distinct separation than the Bray–Curtis distances between samples in BP and WW (Figure S3). In addition, PERMANOVA analysis indicated a significant difference between the bacterial communities from samples in WP and BP, and ZN-S10 application explained 50.3% of bacterial community variations based on Bray–Curtis distances (*p* < 0.05, Figure 3B, Table S3). These results confirm that TPN and ZN-S10 significantly affected the composition of the rhizosphere bacterial community.

We further assessed bacterial relative abundances at the phylum and genus levels. The proportions of Proteobacteria and Bacteroidota were the most dominant phyla among all three sample types (WW, WP, and BP), and Proteobacteria accounted for 58.1% in WW, 53.0% in WP, and 58.6 in BP; the proportions of Bacteroidota were 19.48%, 23.97%, and 25.29% in WW, WP, and BP, respectively (Figure 3D). In addition to Proteobacteria, Bacteroidota, and Firmicutes, the other bacterial phyla in the rhizosphere soil samples showed significant differences (*p* < 0.05) among groups WW, WP, and BP, based on Kruskal–Wallis H test analysis (Figure 3E). Compared to samples in WW, the relative abundances of Verrucomicrobiota and Armatimonadota were increased by 6.16% and 1.05% in WP, respectively, while they decreased by 3.10%, and 0.19% in BP. Nevertheless, the proportions of Patescibacteria, Firmicutes, and Acidobacteriota showed an opposite trend (decreased in WP, and increased in BP), and the rest of the bacterial phyla (Planctomycetota, Abditibacteriota, and Actinobacteriota) showed a downward trend in WP and BP. Based on Kruskal–Wallis H test analysis, there were five bacterial genera in the rhizosphere soil samples that showed significant differences (*p* < 0.05) among groups WW, WP, and BP (Table S4), and the rest of the five bacterial genera showed no significant differences (*p* > 0.05). We speculate that the composition of the rhizosphere bacterial community was changed as a consequence of the application of ZJUP0398-2 or ZN-S10.

### 2.5. Fungal Beta Diversity and Community Composition

Different from the bacterial communities, the fungal community diversity formed two distinct clusters, based on the PCoA of Bray–Curtis analysis, with the samples of WP in a cluster and the samples of WW and BP in a cluster (Figure 4C). Similar results were identified by the beta diversity analysis of Jaccard distances (Figure S4). PERMANOVA analysis of fungal communities indicated a significant difference between WP and BP, and ZN-S10 application explained 46.5% of fungal communities based on Bray–Curtis distances (*p* < 0.05, Table S3). These results revealed that significant changes in the fungal species composition potentially resulted from TPN.

The fungal community compositions varied significantly in all three groups. The fungal phylum level, including Ascomycota, Chytridiomycota, Olpidiomycota, and Rozellomycota, in the rhizosphere soil samples showed significant differences among groups WW, WP, and BP, based on Kruskal–Wallis H test analysis (*p* < 0.05, Figure 4D,E). The most abundant fungal phylum of the samples was *Ascomycota* in all three types of samples, and the relative abundances of Ascomycota accounted for 83.15%, 82.3%, and 96.4% in WW, WP, and BP, respectively. The relative abundance of *Chytridiomycota* in BP samples that were treated with ZN-S10 and ZJUP0398-2 was higher than in WW samples that were treated with only water and WP samples that were only treated with ZJUP0398-2. In addition, inoculation with ZJUP0398-2 increased the relative abundances of Olpidiomycota and Rozellomycota, while the application of ZN-S10 reduced the relative abundances of Olpidiomycota and Rozellomycota. The fungal genus levels in the rhizosphere soil samples also showed significant differences (*p* < 0.05) among groups WW, WP, and BP, except for *Penicillium* and *Meyerozyma* (Kruskal–Wallis H test, *p* < 0.05, Table S4). We speculate that both ZJUP0398-2 and ZN-S10 had obvious impacts on the rhizosphere fungal community composition.

### 2.6. Co-Occurrence Network Analysis

**Figure 5:**
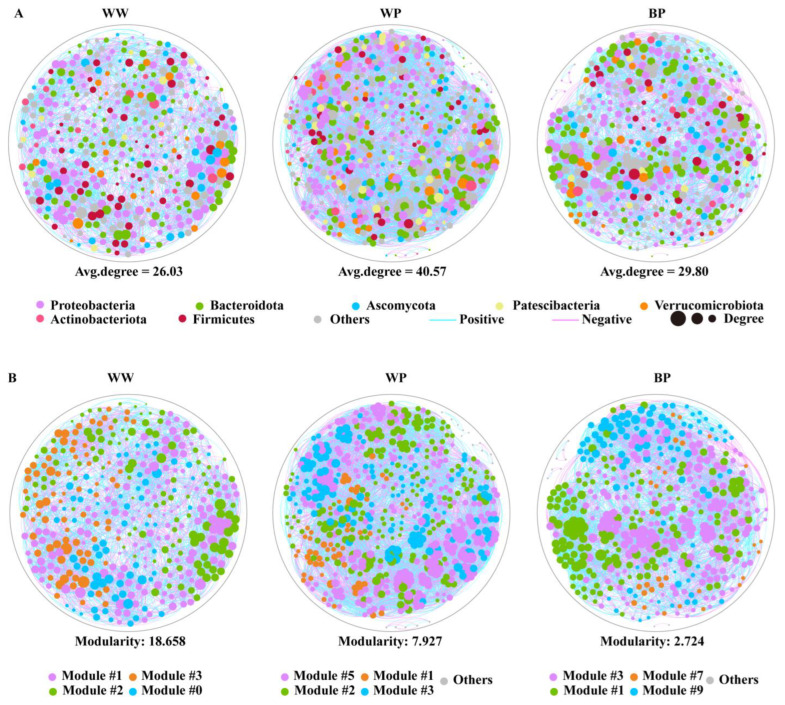
Co-occurrence networks. Co-occurrence network based on the Spearman coefficient correlation of the OTU level. A connection stands for a statistically significant (*p* < 0.05) correlation with a magnitude > 0.6 (positive correlation—blue edges) or < −0.6 (negative correlation—purple edges); different colors represent different phyla (**A**) and distribution of different module classes (**B**).

A co-occurrence network analysis indicated that the composition of the soil microbiomes changed with the applications of ZJUP0398-2 and ZN-S10 (Figure 5A, Tables S5 and S6). Compared with WW, the proportions of the phyla Proteobacteria and Bacteroidota decreased after treatment with ZJUP0398-2 (WP) but increased after the application of ZN-S10 (BP) (Figure 5A). Additionally, the proportion of Firmicutes ranked third in WW, while it ranked sixth in WP and fourth in BP. The ranking of one fungal phylum (Ascomycota) and three bacterial phyla (Patescibacteria, Actinobacteriota, and Firmicutes) also underwent changes.

In the co-occurrence network analysis, network connectivity was the highest for samples in WP. The total nodes and edges in WP were 595 and 12,070, compared with 392 nodes and 5102 edges in WW. A similar trend was observed in BP, in which there were 450 nodes and 6704 edges. In addition, the average degree was increased after inoculation with ZJUP0398-2 (WP). Compared with WP, the application of ZN-10 (BP) resulted in a decrease in the average degree in the presence of ZJUP0398-2. Modularity exhibited a decreasing trend in WW, WP, and BP. Network modularity decreased after inoculation with ZJUP0398-2 in WP. Similarly, the addition of ZN-S10 also decreased the network modularity in BP. We also found that the types of modules with a strong correlation were also gradually concentrated. There were four module classes in WW and WP but three obvious module classes in BP (Figure 5B). These results collectively indicated that the application of ZJUP0398-2 and/or ZN-S10 changed the soil microbial network.

### 2.7. Correlation Analysis of ZN-S10

**Figure 6:**
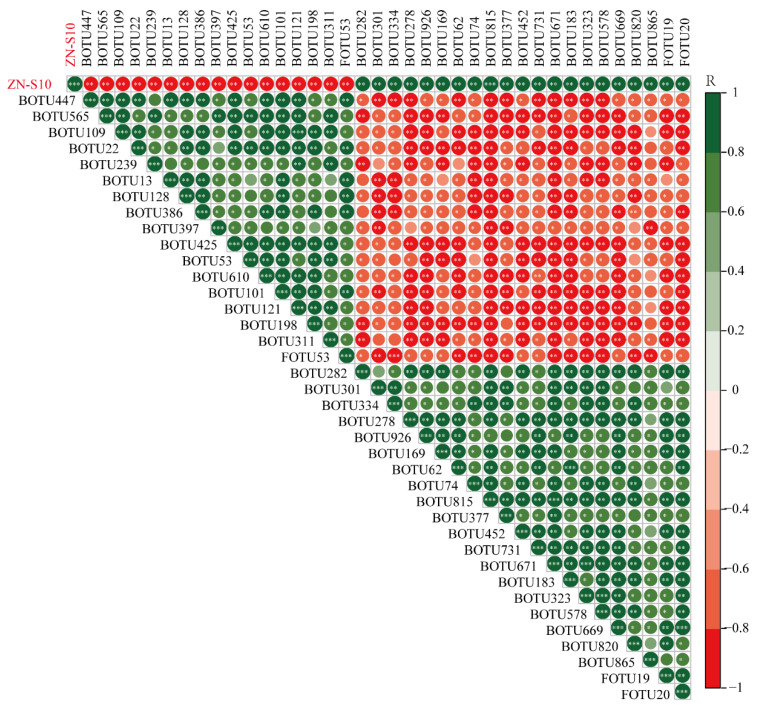
Correlation analysis of ZN-S10. BOTU stands for bacteria, FOTU stands for fungus. Colors represent correlations, with red indicating negative correlation, green indicating positive correlation, and an asterisk representing significance; * *p* < 0.05, ** *p* < 0.01, *** *p* < 0.001.

To further verify the impact of the addition of ZN-S10 on microbial communities, we conducted a correlation analysis (Figure 6, Table S7). We found correlations between ZN-S10 and different bacterial communities, among which Proteobacteria had the largest number of phyla, with a total of 12, and Bacteroidota was in second place, with a total of 7. Correlations between ZN-S10 and fungal communities also existed; two OTUs were positively correlated with ZN-S10, and one was negatively correlated. Notably, the cumulative relative abundances of 29 OTUs belonging to Proteobacteria, Firmicutes, Bacteroidota, Verrucomicrobiota, Abditibacteriota, Armatimonadota, Planctomycetota, Ascomycota, and Actinobacteriota showed significant differences between WP and BP (*p* < 0.05), in which 15 OTUs displayed a decreasing trend, and 14 OTUs displayed an increasing trend. Overall, our data demonstrate a correlation between ZN-S10 and rhizosphere soil microbial communities, especially with OTUs belonging to Proteobacteria and Bacteroidota, which are more strongly correlated. In addition, correlation analysis showed that OTU386 and OTU425 were negatively correlated with ZN-S10 (Figure 6). Taxonomic annotation results revealed that OTU386 was a member of the family Reyranellaceae, and the members belonging to Reyranellaceae may be involved in nitrification [30]; OTU425 classified into the genus *Sphingomonas* functions as a bacterial iron carrier and has a nitrogen-fixing ability [31]. OTU169, which was positively correlated with ZN-S10 (Figure 6, was classified into *Mucilaginibacter*, members of which promote plant growth, induce salt tolerance, and increase cytoplasmic signaling in plants [32]. Additionally, OTU74 belongs to *Chitinophaga* and may be associated with plant disease resistance [33]. Combined with the trend of cumulative relative abundance, it was speculated that the application of ZN-S10 tends to suppress or recruit microorganisms in order to suppress pathogenic microorganisms and/or boost plant immunity.

## 3. Discussion

The members of the *Bacillus* genus are known for their ability to produce dormant spores, enabling them to endure harsh environmental conditions [34]. The FDA regards *Bacillus* as generally safe for food and pharmaceutical uses [35]. Some rhizobacteria have positive impacts on plant growth and are known as plant growth-promoting rhizobacteria (PGPR). Among the well-studied and widely applied PGPR is the *B. velezensis* FZB42 strain. FZB42 possesses the capability to produce a diverse range of antimicrobial compounds, and Chen et al. demonstrated that it can produce non-ribosomal peptides as secondary metabolites to antagonize pathogenic bacteria [36]. The application of *Bacillus amyloliquefaciens* L-S60 has been shown to promote the growth conditions of cucumber seedlings, resulting in the increased availability of mineral elements, compared to the control group [19]. *Bacillus amyloliquefaciens* ARP23 and MEP218 can produce fengycin, iturin, or surfactin, which are effective in the biocontrol of Sclerotinia, a stem rot disease [37]. Chen et al. also revealed that *Bacillus amyloliquefaciens* PG12 can effectively inhibit apple ring rot caused by Botryosphaeria dothidea, with iturin A playing an important role in this activity [38]. In this study, dual-culture assays demonstrated that ZN-S10 could obviously inhibit the growth of the pathogenic bacterium ZJUP0398-2, and pot experiments demonstrated that ZN-S10 had good preventive and control effects on tomato pith necrosis caused by ZJUP0398-2.

Although specific microorganisms can protect plants from soil-borne pathogens, their effectiveness is largely influenced by their interaction with the native microbial communities in the rhizosphere [14]. Therefore, we investigated the changes in the structure of the rhizosphere microbial community of tomato plants caused by the inoculation of ZN-S10 through the analysis of the rhizosphere microbiome. The invasion of plant pathogens can alter the diversity and structure of rhizobacterial communities [19]. We found that the invasion by *P. viridiflava* ZJUP0398-2 significantly altered the richness and diversity of the tomato rhizobacterial community, but pre-inoculation with ZN-S10 mitigated these changes. Research suggests that the more complex the network, the greater the beneficial impact on the microbial community [39,40]. Co-occurrence network analysis revealed that after the *P. viridiflava* ZJUP0398-2 invasion, the average degree increased, indicating a more complex network relationship in the tomato roots. However, after pre-inoculation with ZN-S10, the average degree of the network was similar to that of the water-treated samples, indicating that the use of ZN-S10 maintained the stability of the tomato rhizosphere microbial community and suppressed the occurrence of tomato diseases.

Tomato pith necrosis, a condition that significantly affects tomato yield, is more likely to occur in high-nitrogen environments. Members of the *Sphingomonas* genus are bacteria with iron-carrying properties and nitrogen-fixing capabilities [31]. Studies have shown that increased iron carriers and nitrogen content can promote the pathogenicity of pathogens [41,42]. However, bacteria belonging to the *Mucilaginibacter* genus can help promote plant growth, induce salt tolerance, and enhance cytoplasmic signals in plants [32]. Similarly, species in the genus *Chitinophaga* are associated with plant disease resistance [33]. In this study, we observed a strong negative correlation between ZN-S10 and one OTU classified as *Sphingomonas*, as well as strong positive correlations between ZN-S10 and one OTU classified as *Mucilaginibacter* and one OTU classified as *Chitinophaga*. Based on these findings, we speculate that the application of ZN-S10 maybe inhibits the growth of nitrogen-fixing bacteria and recruits beneficial bacterial communities that are associated with disease resistance, thus suppressing the occurrence of diseases and promoting plant growth.

It is possible that pathogen-infected plant roots can attract beneficial micro-organisms by releasing volatile organic compounds or altering the synthesis and secretion of specific root secretions [43]. *Candidatus Liberibacter* causes decreased relative abundance and/or expression activity of rhizoplane-enriched taxonomic and functional properties [44]. Effective rhizosphere colonization is a crucial step in providing growth-promoting and disease-preventing activities. *B. velezensis* FZB42 colonization triggers a plant immune response and the production of a reactive oxygen species, which stimulates the bacterium to produce a growth hormone, which promotes bacterial survival and effective root colonization [45]. There is growing evidence that plants adapt to biotic stresses (e.g., specific *Bacillus* and *Pseudomonas* spp.) by altering root secretion chemistry, thereby favoring the recruitment of protective plant beneficial microorganisms [46]. For example, l-malic acid secreted by Arabidopsis roots selectively recruits *Bacillus subtilis* FB17 for inter-root colonization [47]. Disruption of the ethylene pathway in tomato leads to changes in root secretions that indirectly regulate the root bacterial community [48]. Therefore, the impact of the secretion of tomato root exudates on rhizosphere microorganisms after treatment with ZN-S10 is an intriguing research direction, which we will delve into.

## 4. Materials and Methods

### 4.1. Experiment Description and Soil Sampling

The greenhouse experiment tomato was Diana F1 purchased from Hebei Maohua Seed Industry Co., Ltd. (Hebei, China). For the bioassays, tomato was grown in a greenhouse and inoculated (or not) with *P*. *viridiflava* and *B*. *velezensis* ZN-S10. Briefly, the seeds were surface-disinfected with a 0.5% (*v*/*v*) sodium hypochlorite solution. In order to minimize differences in soil conditions, the air-dried natural soil (sieved < 4 mm) was autoclaved twice at 120 °C for 20 min. The seeds were germinated in a nutrient substrate; then, the uniform seedlings, of which the roots were rinsed with sterile water, after 14 days, were transplanted into 0.5 L pots containing sterile natural soil. After 28 days of transplantation, the experiment involved three treatment groups: WP, BP, and WW. In the WP group, the roots of the tomato plants were irrigated with sterile water. In the BP group, the roots were irrigated with the ZN-S10 bacterial solution. The WW group received sterile water treatment. After 7 days, both the WP and BP groups were inoculated with *P. viridiflava* in the stem of the tomato plants. However, the WW group was offered sterile water. In this study, the WW group served as the control. Plants were grown in a greenhouse with 24 °C/24 °C day/night temperatures for 16 h/d with 70% relative humidity and were watered weekly with ddH_2_O. The experiment was conducted with 5 sets of tomato plants, each consisting of 10 plants. The plants were kept at a temperature of 24 °C for 16 h per day, with a relative humidity of 70%. They were watered once a week with ddH_2_O (Figure 1).

After 14 days of inoculation with *P*. *viridiflava*, the number of diseased plants was investigated and graded, and records were graded according to the percentage of the wilted portion of the plant to the whole plant. The disease severity was assigned to one of four classes: class 0 = 0%, class 1 = 1–25%, class 2 = 26–50%, class 3 = 51–75%, and class 4 = 76–100%. The disease index (DI) for lily Fusarium wilt incidence was calculated with Equation (1).

DI (%) = 100 × ∑(number of plants assigned to class i × severity class)/(Total leaves surveyed × 4)(1)

The disease reduction (DR; %) was calculated with the formula:

DR(%) = (DI-ck − DI-test)/DI-ck × 100(2)

After 14 days of inoculation with *P*. *viridiflava*, the surface soil was carefully removed using a sterile shovel. Tomato roots were then extracted from the soil, and the root-adhered rhizosphere soil was brushed off using a sterilized brush. The rhizosphere soil was collected in a 50 mL polypropylene microcentrifuge tube and immediately frozen in liquid nitrogen. The samples were then transferred to the laboratory and stored at −80 °C.

### 4.2. Soil DNA Extraction and High-Throughput Sequencing

Total genomic DNA was extracted from the rhizosphere soil using the TGuide S96 Magnetic Soil/Stool DNA Kit (Tiangen Biotech Co., Ltd., Beijing, China) according to the manufacturer’s instructions. The quality and quantity of the extracted DNA were examined using electrophoresis on a 1.8% agarose gel, and DNA concentration and purity were determined with a NanoDrop 2000 UV–Vis spectrophotometer (Thermo Scientific, Wilmington, NC, USA). These DNA extracts were used to amplify the 16S rDNA gene (for bacteria) and the internal transcribed spacer (ITS) rDNA gene (for fungi). The full-length 16S rDNA gene was amplified with primer pairs 27F: AGRGTTTGATYNTGGCTCAG and 1492R: TASGGHTACCTTGTTASGACTT, and the full-length ITS rDNA gene was amplified with primer pairs ITS1F: CTTGGTCATTTAGAGGAAGTAA and ITS4R: TCCTCCGCTTATTGATATGC. The KOD One PCR Master Mix (TOYOBOLife Science Co., Ltd., Shanghai, China) was used to perform 25 cycles of PCR amplification, with initial denaturation at 95 °C for 2 min, followed by 25 cycles of denaturation at 98 °C for 10 s, annealing at 55 °C for 30 s, and extension at 72 °C for 1 min 30 s, with a final step at 72 °C for 2 min. The PCR amplicons were purified using VAHTSTM DNA Clean Beads (Vazyme, Nanjing, China) and quantified with the Qubit dsDNA HS Assay Kit and a Qubit 3.0 Fluorometer (Invitrogen, Thermo Fisher Scientific, Oregon, USA). The amplicons were then pooled in equal amounts, and SMRTbell libraries were prepared using the SMRTbell Express Template Prep Kit 2.0 according to Pacific Biosciences’ instructions. The SMRTbell libraries from the pooled and barcoded samples were purified and sequenced on a PacBio Sequel II platform (Beijing Biomarker Technologies Co., Ltd., Beijing, China) using the Sequel II binding kit 2.0. All library preparation and sequencing steps were performed by Biomarker Technologies (Beijing, China).

### 4.3. Bioinformatics and Statistical Analysis

The bioinformatics analysis of this study was conducted using the BMK Cloud (http://www.biocloud.net/, accessed on 5 April 2023). The raw reads obtained from sequencing were filtered and demultiplexed using SMRT Link software (version 8.0), with the parameters minPasses ≥ 5 and minPredictedAccuracy ≥0.9, to obtain circular consensus sequencing (CCS) reads. The CCS sequences were then assigned to their respective samples based on barcodes using lima (version 1.7.0). CCS reads without primers and those outside the length range (1200–1650 bp) were removed by identifying forward and reverse primers and applying quality filtering through the Cutadapt [49] (version 2.7) quality control process. The UCHIME algorithm (v8.1) [50] was employed to detect and eliminate chimera sequences, resulting in clean reads. Sequences with a similarity > 97% were clustered into the same operational taxonomic unit (OTU) using USEARCH [51] (v10.0), and OTUs with less than 2 counts in all samples were filtered out. The sequences with similarity thresholds above 97% were assigned to an operational taxonomic unit (OTU) using USEARCH (version 10.0). Taxonomy annotation of the OTUs/ASVs was conducted using the Naive Bayes classifier in QIIME2 [52]. The SILVA database was used as the bacterial reference database, and the UNITE database [53] (release 138.1) was used as the fungal reference database. A confidence threshold of 70% was applied. Alpha was performed to identify the complexity of species diversity of each sample using QIIME2 software (v 2020.6.0). Beta diversity calculations were analyzed by principal coordinate analysis (PCoA) to assess the diversity in samples for species complexity. Stacked bar plots of community composition were visualized using the ggplot2 package in R (version 4.3.1). The non-parametric (ranked-based) Kruskal–Wallis test was included to statistically examine differences in alpha diversity between different types of samples. The statistical analyses were conducted in RStudio (v 4.3.1) using Phyloseq, Microbiomeanalyst, and vegan R packages [54,55,56,57,58]. 

### 4.4. Co-Occurrence Network Analysis

Co-occurrence network analysis was performed in R (v 4.3.1) using the Spearman correlation coefficient. According to the strong (R > 0.6) and significant correlations (*p* < 0.05), co-occurrence models within the rhizosphere soil microbial community were constructed. The co-occurrence network was visualized on the Gephi platform using the Fruchtermann–Feingold layout. 

## 5. Conclusions

In the field of agriculture, members of the genus *Bacillus* can be widely applied in crop protection and soil improvement. In this study, *B. velezensis* ZN-S10 significantly inhibited the growth of *P. viridiflava* ZJUP0398-2, and ZN-S10 could effectively control the occurrence of TPN diseases. *P. viridiflava* ZJUP0398-2 significantly altered the richness and diversity of the tomato rhizobacterial community, but pre-inoculation with ZN-S10 mitigated these changes; ZN-S10 may have the potential to attract advantageous microbial communities that enhance disease resistance while simultaneously suppressing microbial communities that promote pathogen growth, thereby suppressing the occurrence of diseases.

## Data Availability

All raw sequence data reads are available at the NCBI SRA under accession numbers SRR25593771-SRR25593785 (BioProject accession number PRJNA1004066 for Bacteria) and SRR25593645- SRR25593659 (BioProject accession number PRJNA1004057 for Fungi).

## Supplementary Materials

The following supporting information can be downloaded at: https://www.mdpi.com/article/10.3390/plants12203636/s1, Figure S1. rarefaction curves and rank abundance curve sparse curves of 15 samples for 16S rDNA gene sequencing. (A) Rarefaction curves of 16S gene sequencing. (B) rank abundance curve sparse curves of 116S gene sequencing; Figure S2. Rarefaction curves and rank abundance curve sparse curves of 15 samples for ITS gene sequencing. (A) Rarefaction curves of ITS gene sequencing. (B) rank abundance curve sparse curves of ITS gene sequencing; Figure S3. Principal coordinate analysis (PCoA) for bacteria using the Abundance-based Jaccard distance matrix. Figure S4. Principal coordinate analysis (PCoA) for Fungi using the Abundance-based Jaccard distance matrix; Table S1: Control efficacy; Table S2: Summary on raw data process; Table S3: PERMANOVA Analysis; Table S4: Kruskal–Wallis H test; Table S5: Correlations networks; Table S6: TOP 10 Phylum in network; Table S7: Correlation analysis of ZN-S10.
