# Supplementary material for: Bacillus velezensis ZN-S10 Reforms the Rhizosphere Microbial Community and Enhances Tomato Resistance to TPN
Source: Plants (Basel). 2023 Oct 21;12(20):3636. doi: 10.3390/plants12203636 (PMC10609795; doi:10.3390/plants12203636)
Supplement: Supplementary file 1 [file plants-12-03636-s001.zip › Supplementary figure.pdf]

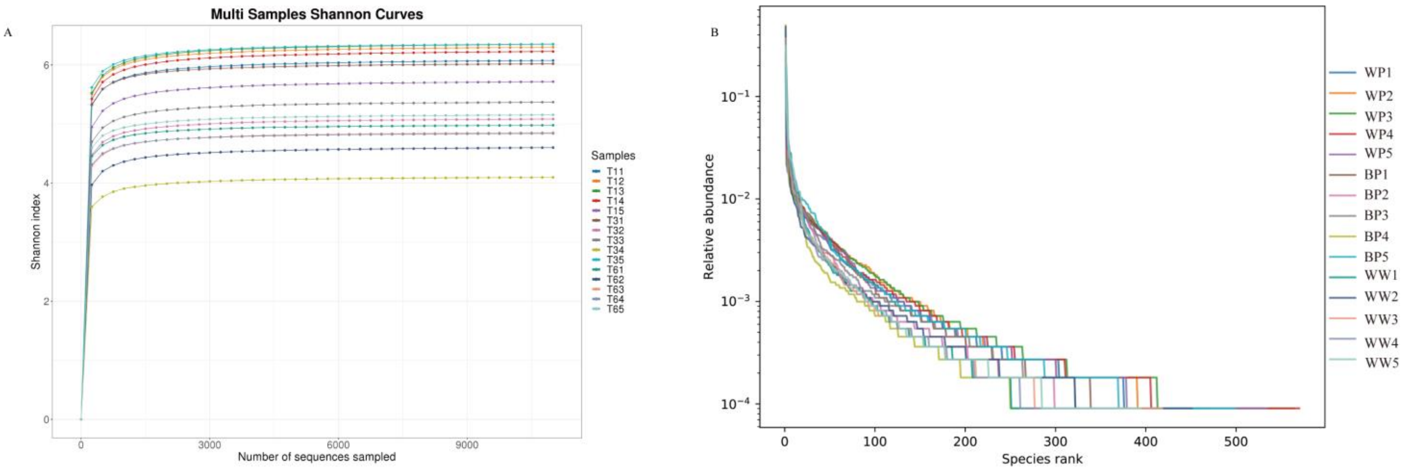

Figure S1. rarefaction curves and rank abundance curve sparse curves of 15 samples for 16S rDNA gene sequencing. A, Rarefaction curves of 16S gene sequencing. B, rank abundance curve sparse curves of 116S gene sequencing.

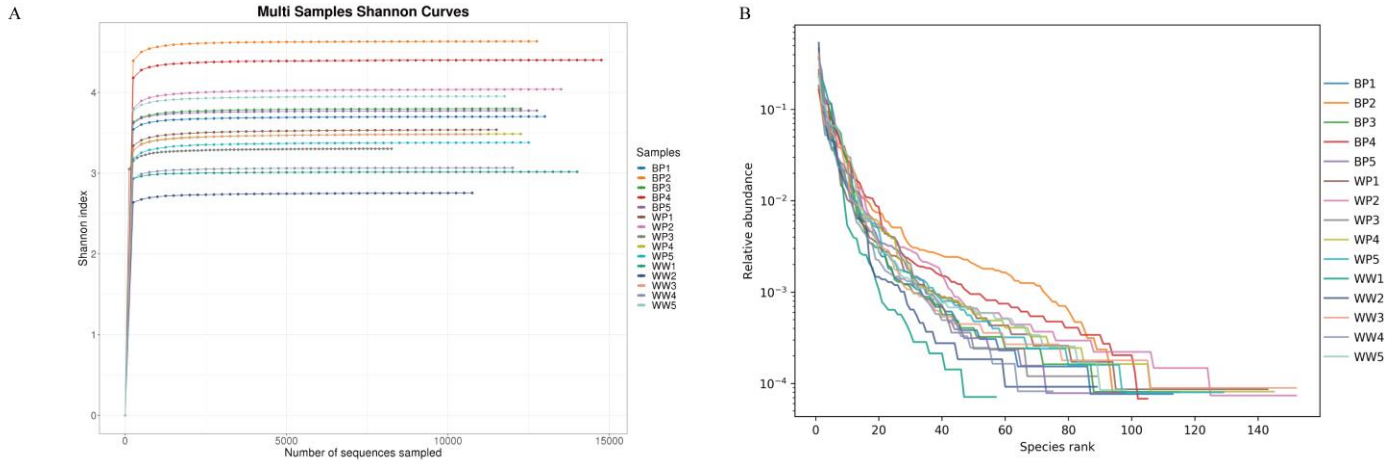

Figure S2. Rarefaction curves and rank abundance curve sparse curves of 15 samples for ITS gene sequencing. A, Rarefaction curves of ITS gene sequencing. B, rank abundance curve sparse curves of ITS gene sequencing.

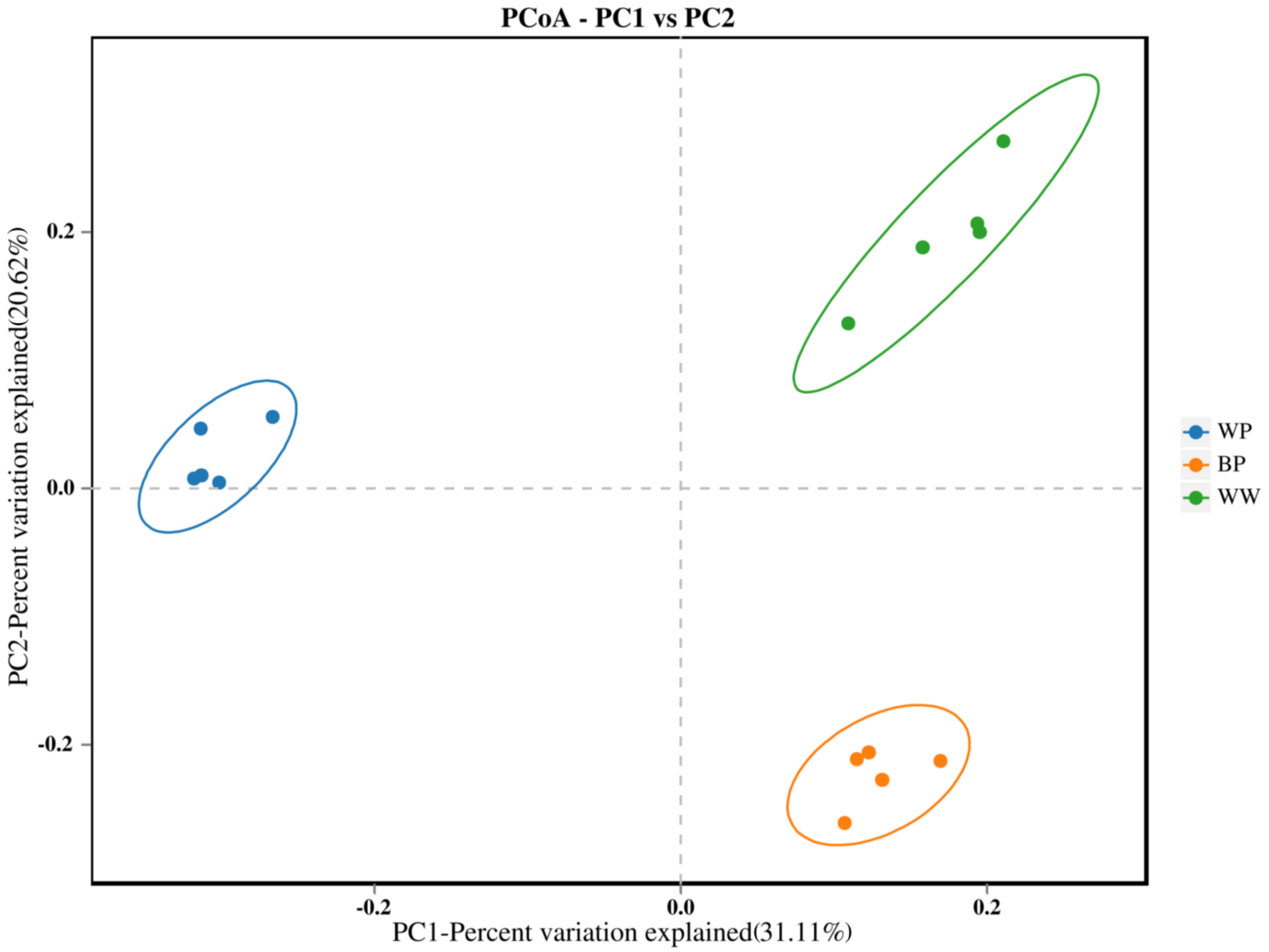

Figure S3. Principal coordinate analysis (PCoA) for bacteria using the Abundance-based Jaccard distance matrix**.**

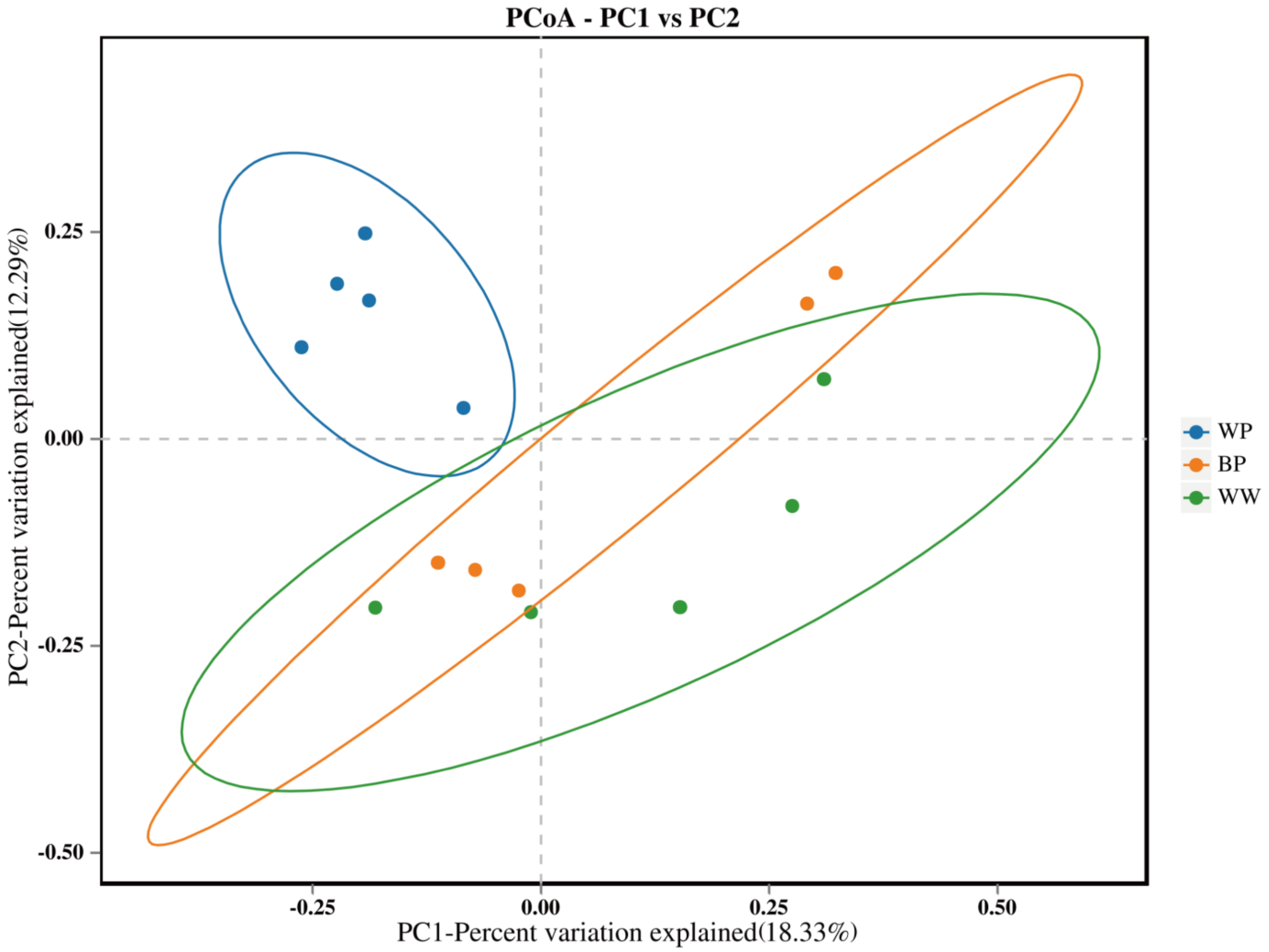

Figure S4. Principal coordinate analysis (PCoA) for Fungi using the Abundance-based Jaccard distance matrix.
