# Supplementary figures and images for: Bacillus velezensis ZN-S10 Reforms the Rhizosphere Microbial Community and Enhances Tomato Resistance to TPN
Source: Plants (Basel). 2023 Oct 21;12(20):3636. doi: 10.3390/plants12203636 (PMC10609795; doi:10.3390/plants12203636)

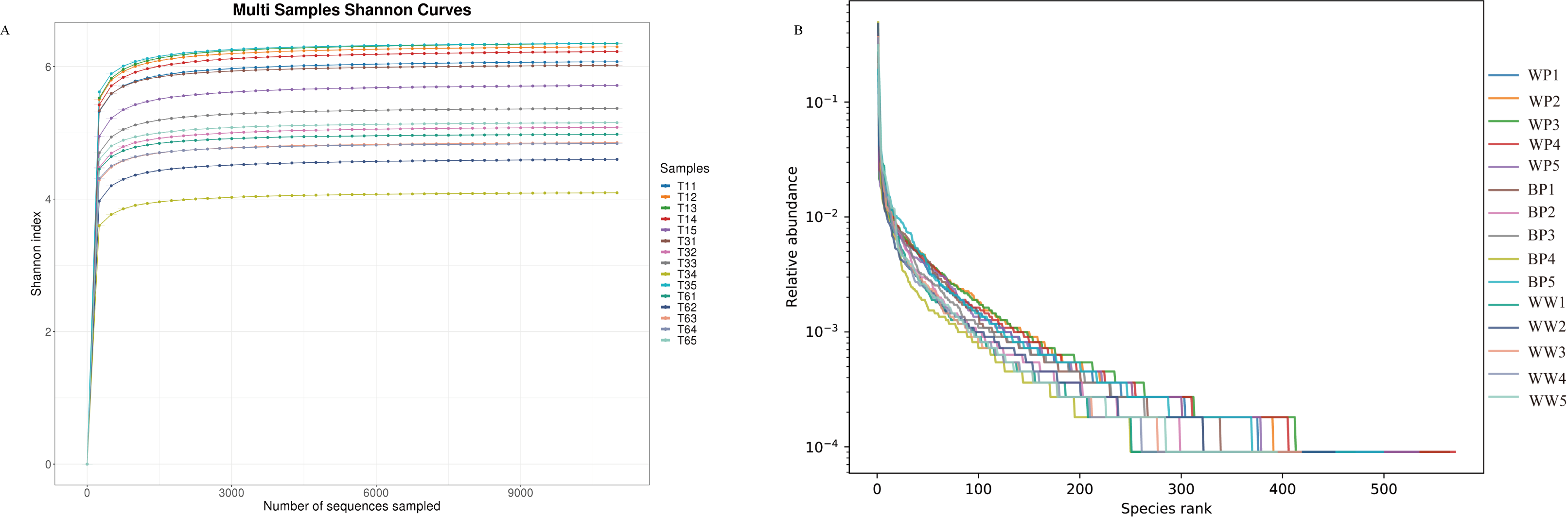

Supplement: Supplementary file 1 [file plants-12-03636-s001.zip › FIG.S1.tif]

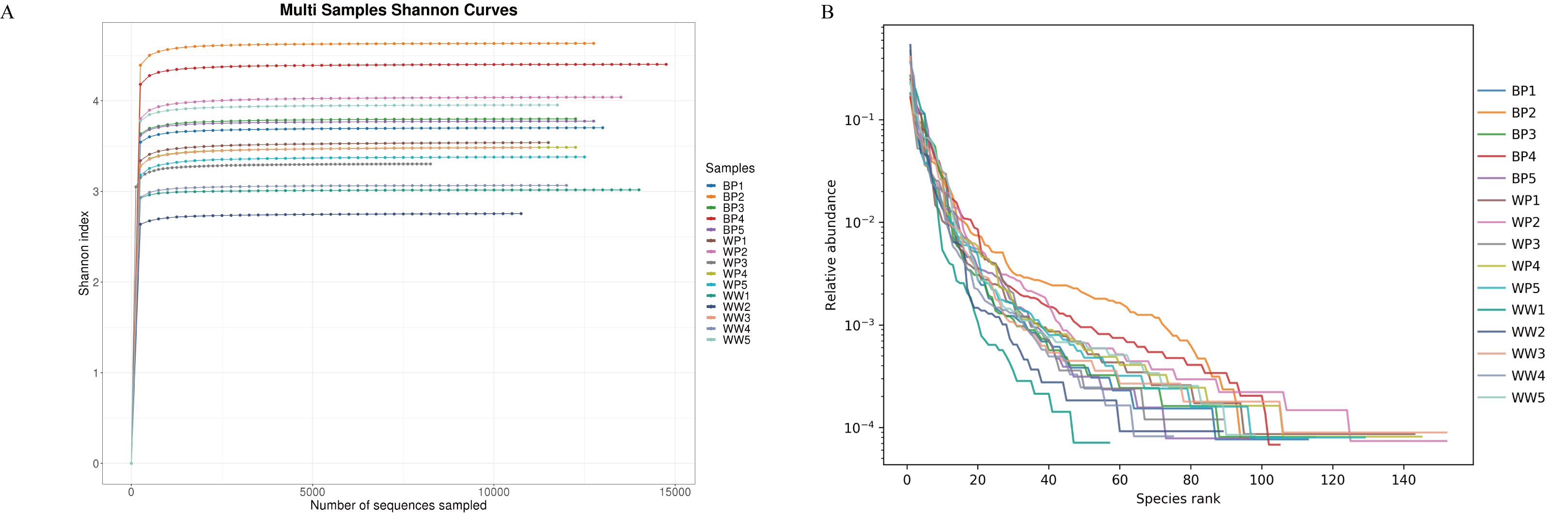

Supplement: Supplementary file 1 [file plants-12-03636-s001.zip › FIG.S2.tif]

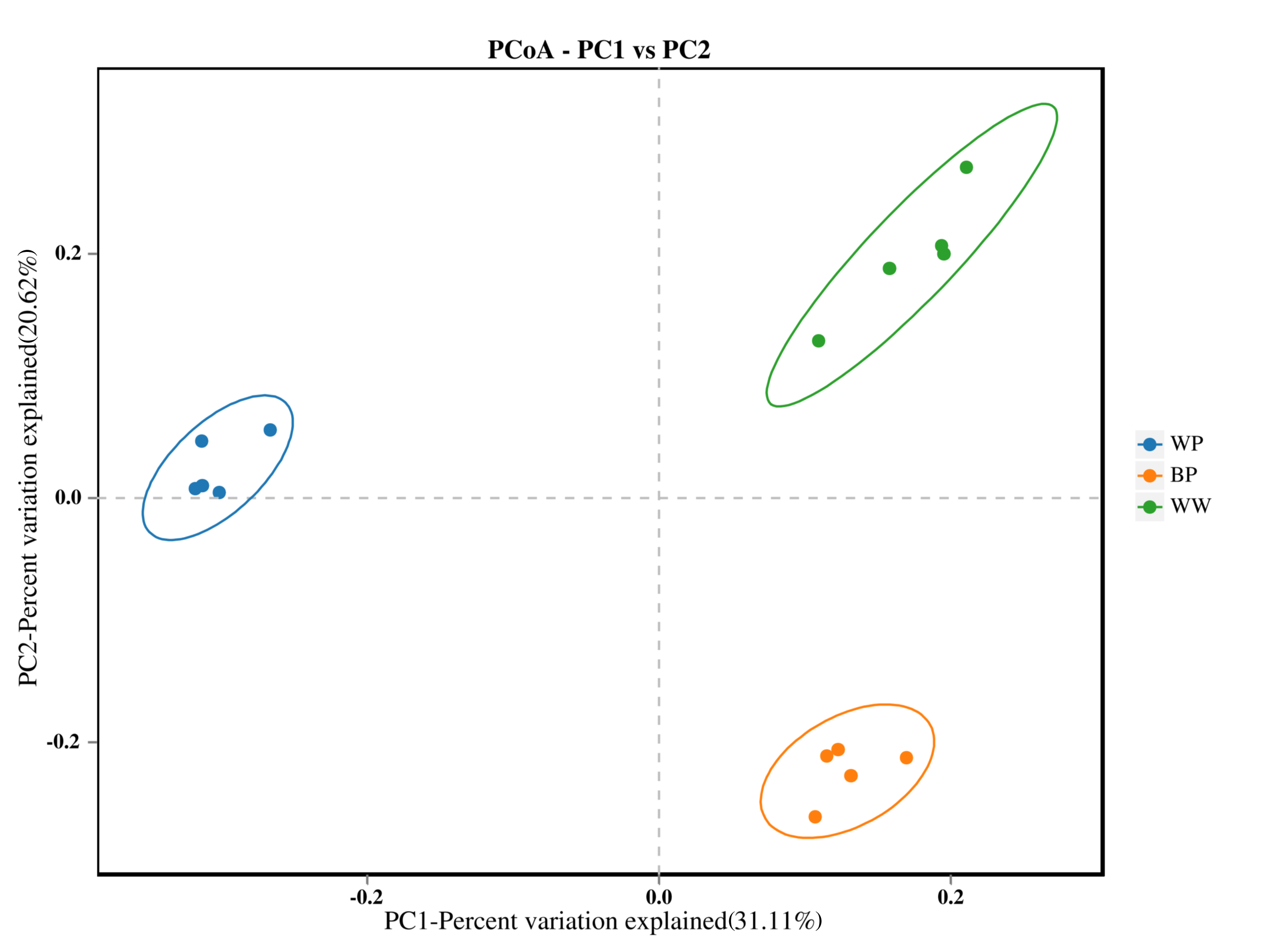

Supplement: Supplementary file 1 [file plants-12-03636-s001.zip › Fig.S3.tif]

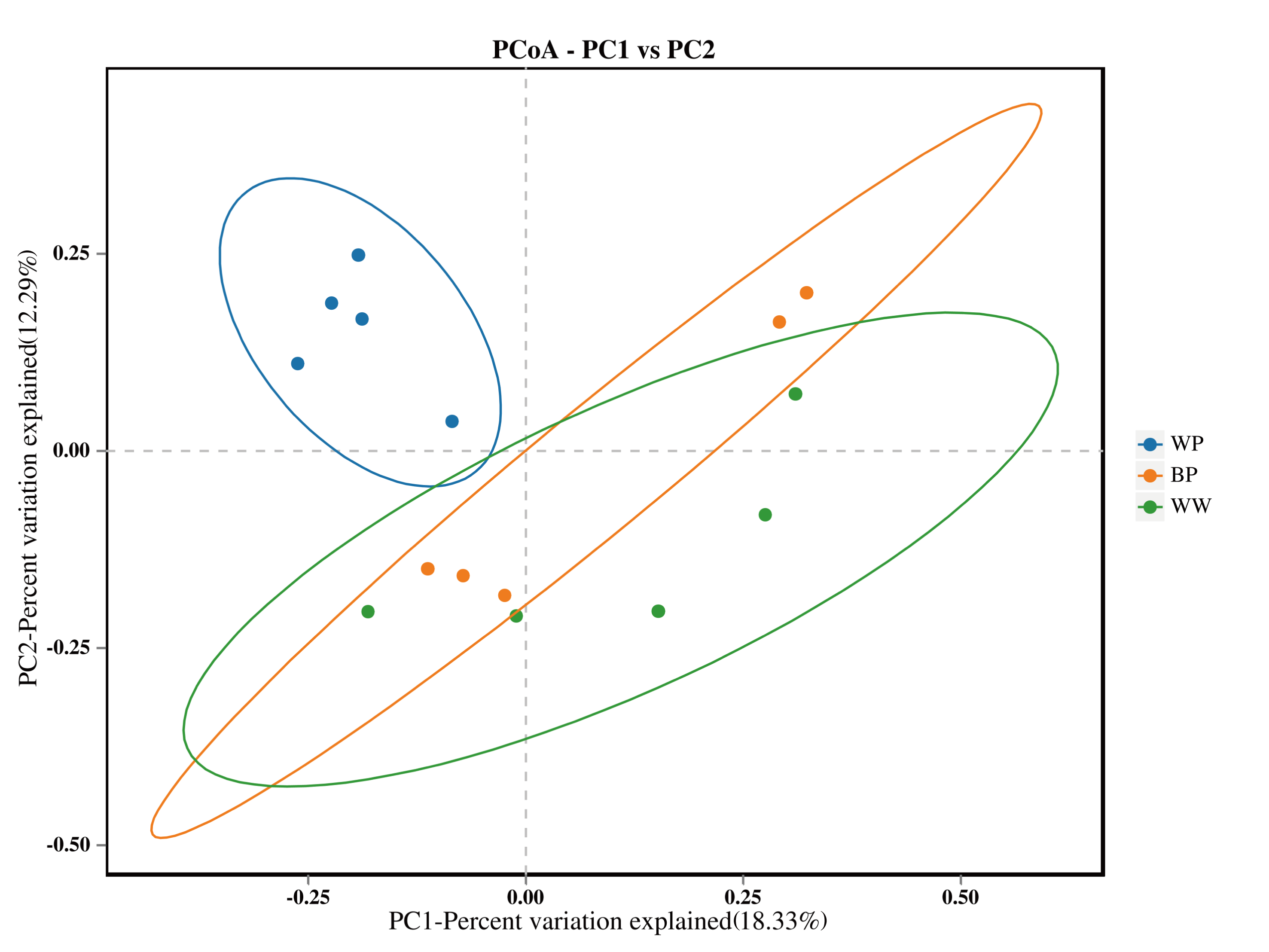

Supplement: Supplementary file 1 [file plants-12-03636-s001.zip › Fig.S4.tif]
